# Identification of Cell Subpopulation-Specific Driver Genes Reveals Ideal Candidates for Renal Cell Carcinoma Immunotherapy

**DOI:** 10.3390/ijms27083467

**Published:** 2026-04-13

**Authors:** Xiangzhe Yin, Lu Wang, Yanwu Sun, Shiyi Li, Wentong Yu, Siyao Wang, Zhichao Geng, Hongying Zhao, Li Wang

**Affiliations:** College of Bioinformatics Science and Technology, Harbin Medical University, Harbin 150081, China

**Keywords:** renal cell carcinoma, gene regulatory network, immune-related candidate driver genes, immunotherapy, tumor microenvironment, single-cell RNA sequencing

## Abstract

With the rapid development of cancer treatment, immunotherapy has revolutionized renal cell carcinoma (RCC) treatment, yet patient responses remain heterogeneous. Here, a computational pipeline was constructed by integrating single-cell and bulk RNA sequencing data to identify immune-related candidate driver genes and characterize their impact on RCC immunotherapy. Based on gene regulatory networks (GRN), 25 immune-related candidate driver genes were identified, leading to the stratification of patients into three clusters (C1–C3). Compared to the C2/C3 cluster, the C1 cluster exhibited elevated immune infiltration, tumor mutation burden and checkpoint expression, which may represent immunotherapy responders. Dynamic analysis of GRNs revealed the critical role of candidate driver genes in predicting the efficacy of immunotherapy. *IRF1*, *IRF9* and *STAT1* in lymphoid cells of C1 participated in anti-tumor immune response by impacting target genes *CD8A*, *HLA-A/E*, *TAP1* and *PD-1*. *JUN*, *FOS*, *STAT3*, *JUND* and *NR2F1* were up-regulated in clusters C2 and C3, leading to tumor progression and immune evasion by influencing target genes *HSPA1A*, *CXCL9* and *PDGFR*. In conclusion, integration of the transcriptome with molecular networks provided a network-based framework to uncover immune-related candidate driver genes for stratifying RCC patients, thereby serving as potential therapeutic targets to improve the outcome of RCC immunotherapy.

## 1. Introduction

Renal cell carcinoma (RCC) is a heterogeneous cancer, accounting for more than 90% of renal cancers [1]. RCC is a common malignancy in both men and women, with each histological subtype exhibiting distinct cellular and tumor biological characteristics [2,3]. The advent of single-cell RNA sequencing (scRNA-seq) has provided critical insights into the cellular composition and transcriptional heterogeneity of RCC. *JAK3* has been associated with immune cell infiltration and can predict response to immune checkpoint inhibitors in RCC [4]. Regulatory T cells can suppress anti-tumor immune responses through *IL-18*, thereby contributing to the maintenance of tumor-promoting immune environments [5]. In recent years, with the study of immune infiltration in tumor microenvironments, immunotherapy strategy has become an effective treatment option for RCC [6].

Although cytokine therapy and adoptive T-cell (ATC) therapy have made great progress, immune checkpoint inhibitors (ICIs) have become a popular immunotherapy method as they enter clinical practice [7]. ICIs include agents that target *CTLA-4*, *PD-1*, and *PD-L1* to promote T-cell effector function and anti-tumor response [8,9]. For example, *CTLA-4* is significantly associated with lymphocyte infiltration and poor survival in patients and is a strong predictor of immunotherapy response [10]. Although therapies targeting *PD-1* and *CTLA-4* have improved the prognosis of RCC patients, there are still significant differences in the immunotherapy outcomes among most patients [11]. Therefore, immune genes play a crucial role in regulating the tumor immune microenvironment and predicting the response to immunotherapy.

In order to predict the therapeutic effect when patients choose immunotherapy strategies, scRNA-seq and bulk RNA-seq data were integrated to analyze the tumor microenvironment of RCC, and a comprehensive analysis was proposed based on cell-specific gene regulatory networks (GRNs). GRNs were constructed to identify immune-related candidate driver genes in tumor cells, myeloid cells, and lymphoid cells. Based on immune-related candidate driver genes, patients were stratified into distinct groups with different predicted responses to immunotherapy. This study will provide strong theoretical evidence for predicting patient immunotherapy outcomes.

## 2. Results

### 2.1. Single-Cell Expression Atlas Unveils Diverse Cell Types in RCC

**Figure 1:**
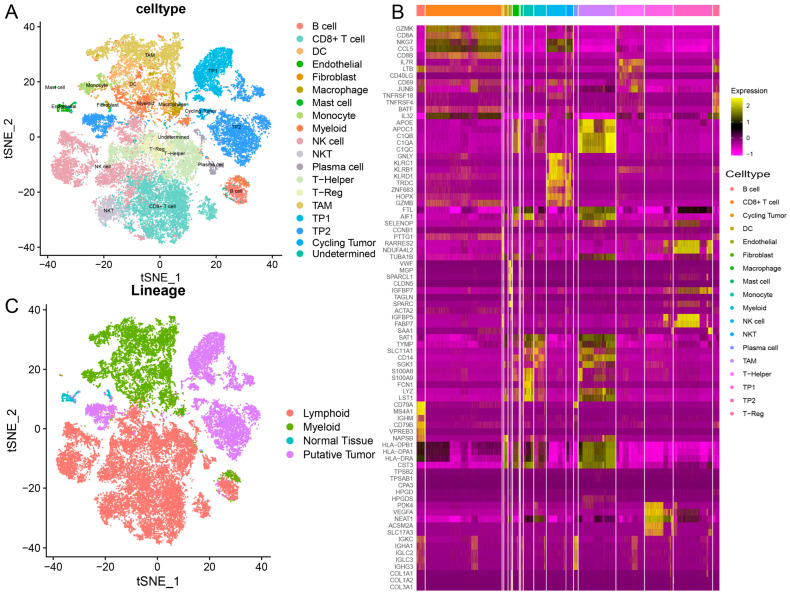
**Single-cell transcriptomics description of the different cell types in RCC.** (**A**) TSNE of malignant and non-malignant cells, colored by cell type. (**B**) The heatmap displays the top 5 significantly differentially expressed genes in each cell type. (**C**) TSNE of cell lineage.

To characterize the tumor microenvironment for RCC, we obtained 34,326 cells from kidney, lymph node, and visceral tissues of eight patients with RCC. Based on clinical outcomes, these patients were stratified into immunotherapy responders (patient identification: P55 and P915) and immunotherapy non-responders (patient identification: P913 and P906). After quality control, 18 distinct cell subsets were identified by known markers, such as CD8+ T cells, B cells, natural killer cells, and macrophages (Figure 1A). Subsequently, differential expression analysis was performed to identify markers for these cell subsets. CD8+ T cells expressed high levels of *GZMK*, *CD8A* and *CD8B*. *TNFRSF18*, *TNFRSF4* and *BATF*, which are specific markers for regulatory T cells, were expressed at high levels in regulatory T cells. *AIF1*, *C1QC* and *C1QB*, as macrophage markers, were specifically up-regulated in macrophages (Figure 1B). Additionally, 8318 malignant cells and 362 normal epithelial cells were distinguished based on copy number variations. Then, unsupervised clustering revealed three distinct tumor subpopulations. Subpopulation TP1 highly expressed *PDK4*, *VEGFA* and *NEAT1*. Subpopulation TP2 had high expression of *CRYAB*, *CLU* and *SEZ6L2*. The cycling tumor subpopulation up-regulated *CCNB1*, *PTTG1* and *TUBA1B* [12]. In summary, these results indicate that there are spatially and transcriptionally distinct cell subsets in RCC patients, including 17,298 lymphoid cells, 8348 myeloid cells, 8318 tumor cells and 362 normal tissue cells (Figure 1C).

### 2.2. Single-Cell Network Inference to Identify Potential Immune-Related Candidate Driver Genes of RCC

**Figure 2:**
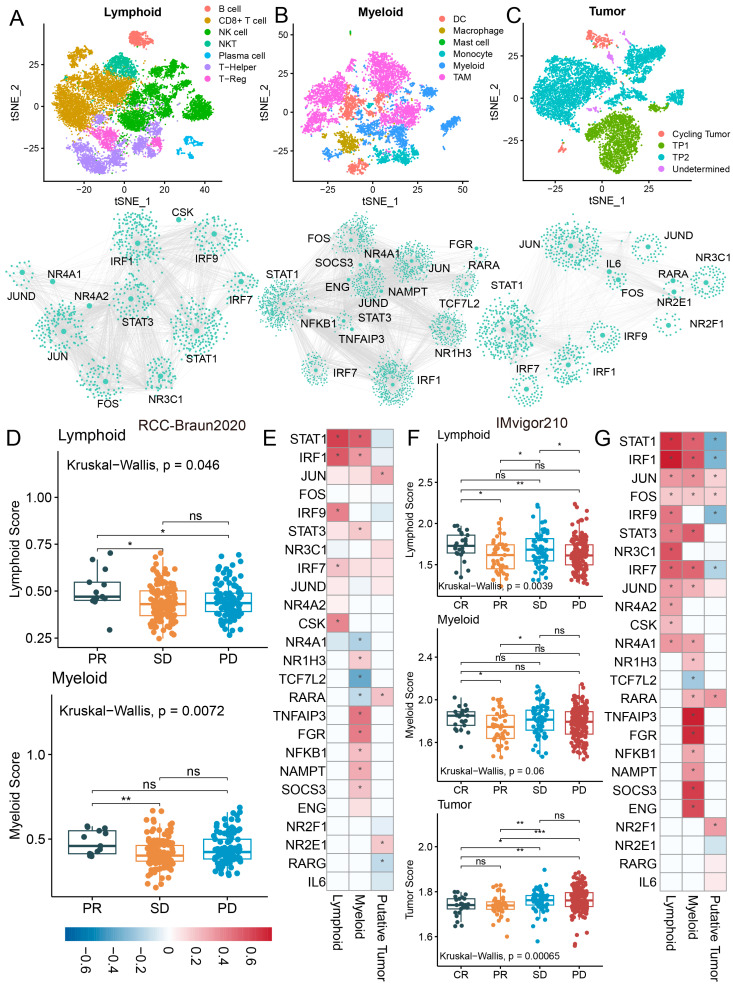
**Identifying immune-related candidate driver genes in RCC.** (**A**–**C**) TSNE and gene regulatory networks of lymphoid cells, myeloid cells, tumor cells. (**D**) The infiltration levels of lymphoid cells and myeloid cells in the immunotherapy cohort (RCC-Braun2020). (**E**) The correlation between immune-related candidate driver genes and infiltration of corresponding cell clusters in the RCC-Braun2020 cohort. (**F**) The infiltration levels of lymphoid cells, myeloid cells and tumor cells in the immunotherapy cohort (IMvigor210). (**G**) The correlation between immune-related candidate driver genes and infiltration of corresponding cell clusters in the IMvigor210 cohort. CR, complete response; PR, partial response; SD, stable disease; PD, progressive disease. ns: not significant; * *p* < 0.05, ** *p* < 0.01, *** *p* < 0.001.

To determine potential driver genes in shaping gene regulatory networks and disease mechanisms for RCC, SCENIC was first used to construct GRNs in lymphoid cells, myeloid cells, and tumor cells, respectively (Figure 2A–C). Next, based on the topological coefficients of GRNs (degree, betweenness, eigenvalue, PageRank, and closeness), the Q statistic was computed for each node in the network. Given the critical roles of immune genes in immune regulation and immunotherapy, those with a top 5% Q statistic were considered as immune-related candidate driver genes from a set of 2013 immune-related genes obtained from the ImmPort database. As a result, a lymphoid-cell-associated GRN was constructed in the lymphoid cell environment, containing 9050 TF–target pairs and 161 regulons. Twelve immune-related candidate driver genes were identified, including nine transcription factors (TF) and three target genes (Figure 2A and Figure S1A). Functional enrichment analysis showed that immune-related candidate driver genes were related to *PD-L1* expression and the *PD-1* checkpoint pathway in cancer, Th1 and Th2 cell differentiation, and the type I interferon signaling pathway (Figure S2A). A myeloid-cell-associated GRN was established in the myeloid cell environment, containing 9988 TF–target pairs and 130 regulons. Seventeen immune-related candidate driver genes were obtained, including eleven TFs and six target genes (Figure 2B and Figure S1B). Functional enrichment analysis showed that driver genes were related to functions and pathways such as regulation of myeloid cell differentiation, Th17 cell differentiation and regulation of innate immune response (Figure S2B). A tumor-cell-associated GRN was constructed in the tumor cell environment, containing 8710 TF–target pairs and 182 regulons. Thirteen immune-related candidate driver genes were obtained, including ten TFs and three target genes (Figure 2F and Figure S1C). Functional enrichment analysis showed that driver genes were related to immune response and tumor-progression-related functions such as the Toll-like receptor signaling pathway, apoptosis and interferon gamma-mediated signaling pathway (Figure S2C). Subsequently, genome-wide CRISPR-Cas9 screening data from the Cancer Dependency Map (DepMap) database were utilized to evaluate the functional impact of immune-related candidate driver genes on cell viability [13,14,15]. Gene effect scores indicated that the knockdown of 12 immune-related candidate driver genes influenced cell viability in the majority of RCC cell lines (Figure S2D). For example, *JUN*, *NR4A1*, *NAMPT*, *JUND* and *SOCS3* depletion markedly suppressed the proliferation in more than 80% of RCC cell lines. These results provide evidence supporting the potential roles of immune-related candidate driver genes in regulating RCC development.

Hence, a total of 25 immune-related candidate driver genes were identified as key factors for RCC development and immune status. Among these, six immune-related candidate driver genes (including *STAT1*, *IRF1*, *JUN*, *FOS*, *IRF7* and *JUND*) were shared by tumor, myeloid and lymphoid cell lineages and eleven immune-related candidate driver genes were common to at least two of the three cell lineages. We then used a single-sample gene set enrichment analysis (ssGSEA) algorithm to assess the infiltration of three cell lineages in an RCC immunotherapy cohort (RCC-Braun2020) and to calculate the correlation with immune-related candidate driver genes. Lymphoid and myeloid cell infiltration showed significant differences among patients in different immunotherapy response groups (*p* < 0.05; Figure 2D). In particular, lymphoid and myeloid cell infiltration were higher in PR patients compared to SD/PD patients. The correlation analysis showed that 72% (18/25) of immune-related candidate driver genes showed significant correlation with the infiltration level of at least one of the three cell lineages (Figure 2E). For example, *STAT1*, *IRF1* and *CSK* were up-regulated in PR patients and were significantly positively correlated with immune cell infiltration (Figure 2E and Figure S1D). *JUN*, *FOS* and *IL6* were up-regulated in non-response (NR) patients (Figure S1E,F). Recent studies have shown that *STAT1*, *IRF1* and *CSK* are linked to immune response [16,17,18], whereas *JUN*, *FOS* and *IL6* are associated with tumor progression [19,20]. In addition, a similar conclusion was drawn from additional validation analysis conducted on a bladder cancer immunotherapy cohort (IMvigor210). Lymphoid and myeloid cell infiltration were higher in CR patients compared to SD/PD patients. However, tumor cell infiltration was significantly lower in CR/PR patients compared to SD/PD patients (Figure 2F). Eighty-eight percent (22/25) of immune-related candidate driver genes showed significant correlation with the infiltration level of the cell lineage (Figure 2G). In summary, immune-related candidate driver genes are involved in the tumorigenesis and immune status of RCC and may serve as candidate biomarkers for predicting the response to immunotherapy in patients.

### 2.3. Immune-Related Candidate Driver Genes Aid in Predicting the Immunotherapy Effect in Patient Clusters

**Figure 3:**
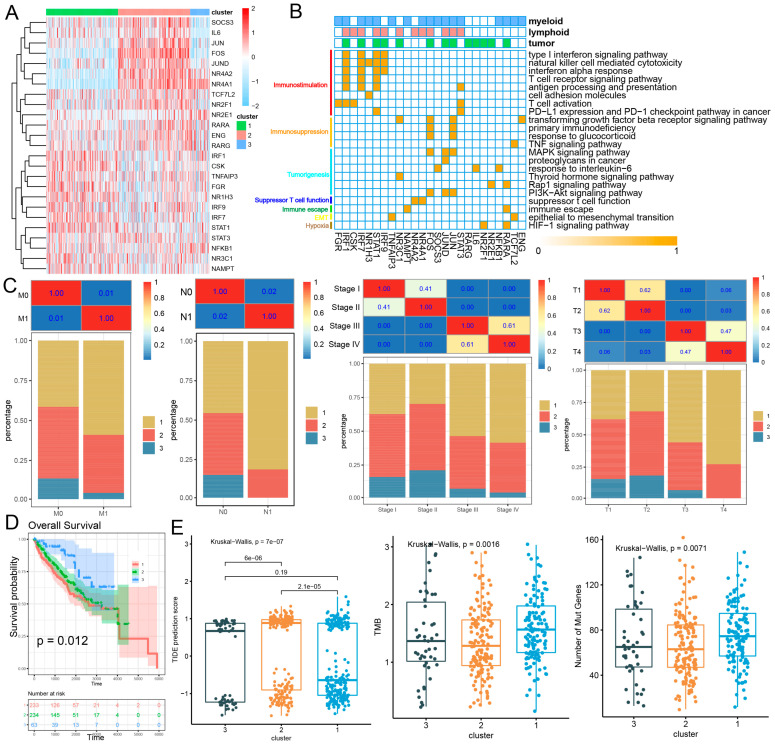
**Identification of potential immunotherapeutic patients of RCC.** (**A**) Heatmap showing the expression of immune-related candidate driver genes across the three groups. (**B**) Functional enrichment analysis of immune-related candidate driver genes. (**C**) The difference in clinical characteristics among C1–C3 groups in TCGA-KIRC cohort. (**D**) Kaplan–Meier curves showed that patients in cluster C1 had poorer OS than those in clusters C2 and C3 in TCGA-KIRC cohort. (**E**) Comparisons of TIDE scores, TMB, and the number of mutation genes across C1, C2 and C3 groups. The Kruskal–Wallis test, Wilcoxon rank sum test and chi-square test were used for statistical significance.

Since immune-related candidate driver genes play an important driving role in the tumor immune microenvironment, we attempted to identify appropriate patient clusters for immunotherapy based on immune-related candidate driver genes. Therefore, unsupervised consensus clustering analysis was used to identify patient clusters of the TCGA-KIRC cohort based on the expression patterns of 25 immune-related candidate driver genes. Based on their cumulative distribution function curves of the consensus score and function delta area, we divided the patients into three clusters (C1–C3; Figure S3A). Among them, *STAT1*, *IRF1*, *IRF7*, *NR1H3*, *FGR* and *CSK* were significantly up-regulated in patients with the C1 group, which were related to the T-cell receptor and type I interferon signaling pathway and antigen presentation and processing (Figure 3A,B and Figure S3B). Immune-related candidate driver genes such as *JUN*, *FOS*, *JUND*, *NR4A2*, *NR4A1*, *IL6*, *SOCS3*, *RARA*, *NFKB1* and *NR2F1* were significantly up-regulated in patients in the C2 or C3 group (Figure 3A and Figure S3B). These genes were related to immunosuppression and suppressor T-cell function to induce immunosuppression and promote tumor development, such as regulation of the TGF-β signaling pathway, response to glucocorticoid, EMT, immune escape and hypoxia. These results suggest that immune-related candidate driver genes define distinct patient patterns, including the immune activation program (C1 group) and the immunosuppressive, tumor progression program (C2 and C3 groups).

Subsequently, the clinicopathological features of patients in the three groups were compared. There were differences in numbers of patients by clinical stage (I–IV), pathologic T (T1–T4), pathologic N (N0, N1), and pathologic M (M0, M1) among the three patient groups. Most patients in M1 and N1 stages were found in C1, while most patients in M0 and N0 stages were in C2 and C3. Most patients with stage I and II disease were in group C2, while most patients with stage III and IV disease were in group C1. Most patients with stage T1 and T2 disease were in group C2, while most patients with stage T3 and T4 disease were in group C1 (Figure 3C). Kaplan–Meier survival analysis indicated that patients in the C1 group presented significantly worse overall survival than those in the C2 and C3 groups (*p* = 0.01; Figure 3D). We predicted the potential response of the three patient clusters to immunotherapy through the tumor immune dysfunction and exclusion (TIDE) algorithm and tumor mutation burden (TMB). A high TIDE score and low TMB indicate a poor response to immune checkpoint blockade. As a result, patients in the C1 group had a significantly lower TIDE score, higher TMB and higher number of mutant genes compared with patients in C2 and C3 groups, which indicated that patients in the C1 group may derive greater benefit from immunotherapy (Figure 3E). Using the GSE167573 cohort as a validation set, an intra-group proportion (IGP) analysis was performed to confirm the agreement and reproducibility of patient groups between the two cohorts. Consistent with the results of TCGA cohort, there were significant differences in prognosis and TIDE scores among different patient groups (Figure S3C,D). Next, the enrichment scores of the C1–C3 groups were calculated from the scRNA-seq data. Immunotherapy-responsive patients (P55 and P915) showed significantly higher enrichment scores in the C1 group, while immunotherapy non-responsive patients (P913 and P906) had significantly higher enrichment scores in C2 and C3 groups, respectively (Figure S3E). Because the immune checkpoint (ICP) genes and immunogenic cell death (ICD) modulators are crucial in the response to immunotherapy, the expression differences of 24 ICD genes and 49 ICP genes among C1–C3 groups were compared in TCGA cohort. Fifteen (62.5%) ICD modulators were significantly differentially expressed among the C1–C3 groups (Figure S3F). For example, the C1 group had high levels of *CALR* (FDR = 2.42 × 10^−6^), *CXCL10* (FDR = 7.37 × 10^−7^) and *FPR1* (FDR = 1.52 × 10^−6^). Forty (81.6%) ICPs were significantly differentially expressed among patient clusters (Figure S3G). For example, major inhibitory ICP genes were significantly up-regulated in the C1 patient group, such as *CTLA-4* (FDR = 3.72 × 10^−7^), *LAG3* (FDR = 1.82 × 10^−9^), *BTLA* (FDR = 7.18 × 10^−9^) and the ligand for *BTLA* (*TNFRSF14*). The overexpressed ICPs were reported to suppress the anti-tumor immune response in the TME [21]. The clinical relevance of C1–C3 groups was evaluated in two immunotherapy-treated cohorts (RCC-Braun2020 and IMvigor210). In both cohorts, patients with immunotherapy response (PR or CR) exhibited significantly higher C1 scores, suggesting an association between the C1 cluster and favorable treatment response (Figure S4A). In contrast, higher C2 scores were consistently associated with worse survival (Figure S4B). Survival analysis further revealed that patients with a high C1 score combined with a low C2 score showed significantly improved survival following anti-PD-L1 therapy, whereas those with a high C2 score and low C1 score had significantly poorer outcomes (Figure S4C). Furthermore, high C1 and low C3 scores were associated with better survival, while the opposite pattern indicated worse prognosis. Taken together, these findings indicate that the C1 group represents more aggressive tumors that have a poor prognosis in the absence of treatment but might be responsive to immunotherapy.

### 2.4. Cellular and Molecular Characteristics of Patient Clusters

**Figure 4:**
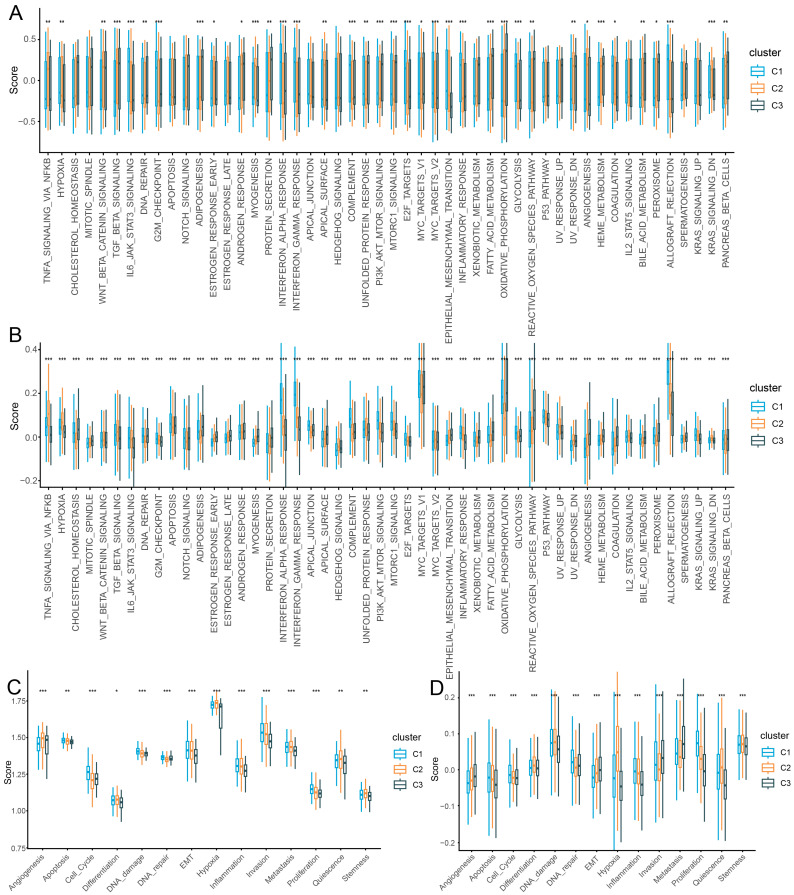
**Functional analysis of different patient clusters.** (**A**,**B**) Significantly enriched gene set variation analysis of Hallmark gene sets between patient clusters in TCGA cohort (**A**) and scRNA-seq dataset (**B**). (**C**,**D**) Distribution of 14 key functional states across the three patient clusters in TCGA cohort (**C**) and scRNA-seq dataset (**D**). The Kruskal–Wallis test was used for statistical significance. The false discovery rate (FDR) correction was applied to correct the *p*-value. ***: FDR < 0.001; **: FDR < 0.01; *: FDR < 0.05.

The antagonistic or promoting effects of the tumor microenvironment on immunotherapy prompted us to explore the cellular and molecular characteristics of patient clusters. Significant differences were observed in the cell subpopulations enriched in the C1, C2, and C3 groups. The C1 group showed significantly higher lymphoid and myeloid cell infiltration, especially CD8+ T cells, natural killer T cells, regulatory T cells, and tumor-associated macrophages. The C2 group showed significantly higher cell infiltration of B cells, T-helper cells and plasma cells. The C3 group showed significantly higher tumor cell infiltration especially for the TP2 tumor subpopulation (permutation test *p* < 0.05; Figure S5). Gene set variation analysis in TCGA cohort and scRNA-seq data showed that C1 was significantly enriched in interferon alpha/gamma response, inflammatory response, complement, G2M checkpoint, and the IL6/JAK/STAT3 signaling pathway (Figure 4A,B). The C2 group was mainly associated with angiogenesis, TNFA signaling via NFKB, estrogen response, the TGF-β signaling pathway, and the KRAS signaling pathway. The C3 group was significantly enriched in metabolism-related functions such as peroxisome, bile acid metabolism, oxidative phosphorylation, fatty acid metabolism, and xenobiotic metabolism. The analysis of the CancerSEA functional states in the C1–C3 patient clusters showed that patient clusters displayed distinct functional states. The C1 group was correlated with several important functional states such as cell cycle, proliferation, inflammation, and DNA repair. The C2 group was correlated with hypoxia, quiescence, and stemness. The C2 and C3 groups were correlated with angiogenesis (Figure 4C,D). Collectively, these results indicate that the three patient groups exhibit distinct cellular compositions and molecular programs, with C1 characterized by an immune phenotype, C2 by a hypoxia- and angiogenesis-associated state, and C3 by a metabolism phenotype.

### 2.5. The Role of Dynamic Regulation of Immune-Related Candidate Driver Genes in Tumor Immunotherapy

**Figure 5:**
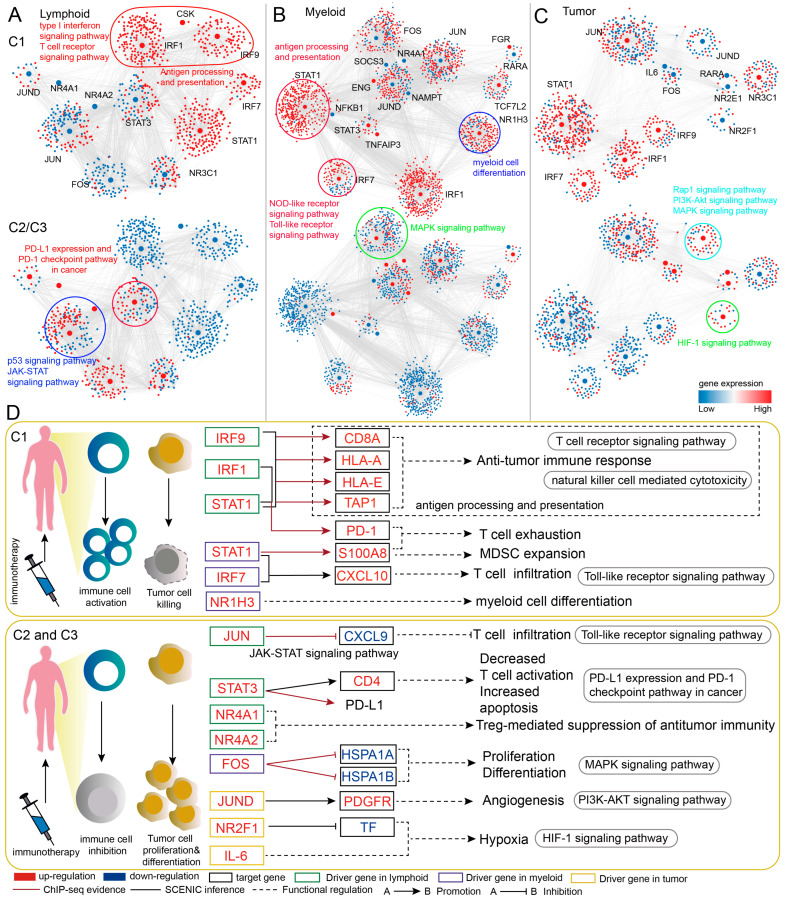
**Gene regulatory network reveals dynamic gene regulatory changes among immunotherapy patient clusters.** (**A**) Dynamic changes in the expression pattern of GRN between clusters in lymphoid cells. The result of differential expression analysis is shown: *IRF1*: FDR = 7.79 × 10^−15^ and log2FC = 0.26; *IRF9*: FDR = 9.80 × 10^−51^ and log2FC = 1.13; *STAT1*: FDR= 8.17 × 10^−122^ and log2FC = 1.98; *CD8A*: FDR = 5.93 × 10^−258^ and log2FC = 1.38; *HLA-A*: FDR < 0.001 and log2FC = 0.78; *HLA-E*: FDR = 1.16 × 10^−68^ and log2FC = 0.34; *TAP1*: FDR = 8.68 × 10^−122^ and log2FC = 1.33; *PDCD1*: FDR = 1.37 × 10^−139^ and log2FC = 1.86; *JUN*: FDR = 2.86 × 10^−87^ and log2FC = 0.98; *CXCL9*: FDR = 1.92 × 10^−21^ and log2FC = −3.41; *CD4*: FDR = 6.49 × 10^−5^ and log2FC = 0.87; *NR4A1*: FDR = 4.55 × 10^−74^ and log2FC = 1.80; *NR4A2*: FDR = 8.40 × 10^−113^ and log2FC = 1.65. (**B**) Dynamic changes in the expression pattern of GRN between clusters in myeloid cells. The result of differential expression analysis is shown: *STAT1*: FDR = 4.05 × 10^−181^ and log2FC = 2.09; *IRF7*: FDR = 3.59 × 10^−41^, log2FC = 0.21; *CXCL10*: FDR = 6.15 × 10^−100^ and log2FC = 3.41; *S100A8*: FDR = 1.41 × 10^−22^ and log2FC = 0.90; *NR1H3*: FDR = 6.34 × 10^−34^ and log2FC = 1.60; *FOS*: FDR = 3.65 × 10^−41^ and log2FC = 1.39; *HSPA1B*: FDR = 6.62 × 10^−61^ and log2FC = −0.48; *HSPA1A*: FDR = 1.64 × 10^−67^ and log2FC = −0.36. (**C**) Dynamic changes in the expression pattern of GRN between clusters in tumor cells. The result of differential expression analysis is shown: *IL-6*: FDR = 1.65 × 10^−8^ and log2FC = 5.59; *TF*: FDR = 4.79 × 10^−15^ and log2FC = −1.34; *JUND*: FDR = 1.13 × 10^−10^ and log2FC = 2.13; *PDGFR*: FDR = 0.0074, log2FC = 2.12. (**D**) Immune-related candidate driver genes affect multiple important biological pathways. The red font indicates an up-regulated gene, while the blue font indicates a down-regulated gene. The black frame represents the target gene, the green frame represents the driver gene in GRN of lymphoid cells, the purple frame represents the driver gene in GRN of myeloid cells, and the yellow frame represents the driver gene in GRN of tumor cells. Solid arrows indicate activation, whereas bar-headed lines denote inhibition. Black solid lines represent TF-target gene regulatory relationships inferred by SCENIC, while brown solid lines indicate those supported by ChIP-seq data. Black dashed lines represent regulatory associations between genes and functions.

In order to elucidate the role of immune-related candidate driver genes in tumor immunotherapy, we characterize the driver-gene-mediated dynamic regulation across different patient groups. Compared with the C2/C3 group, GRNs of lymphoid, myeloid and tumor cells in the C1 patient group showed the expression changes of a large number of TFs and target genes (Figure 5A,C). In the lymphoid-cell-associated GRN, patients in the C1 group showed increased activity of *IRF1* (FDR < 0.001), *IRF9* (FDR < 0.001), and *STAT1* (FDR = 1.16 × 10^−260^) regulons (Figure S6A,B). The up-regulated *IRF1*, *IRF9*, and *STAT1* in the C1 group affected antigen presentation and processing by activating the expression of target genes *CD8A*, *HLA-A*, *HLA-E* and *TAP1* (Figure 5D), potentially promoting the anti-tumor immune response [22]. Increased *STAT1* and *IRF1* expression in the C1 group was associated with elevated expression of *PDCD1*, which may reflect increased *PD-1* expression on exhausted CD8+ T cells. Patients in the C2/C3 group displayed increased activity and expression levels of *JUN*, *STAT3* regulons and up-regulation of *NR4A1* and *NR4A2* to influence *PD-L1* expression and the *PD-1* checkpoint pathway in cancer, the p53 signaling pathway, and the JAK-STAT signaling pathway. In the C2/C3 group, the increased expression of *JUN* may decrease the target gene *CXCL9*, which may inhibit the infiltration of T cells [23]. The high expression of *STAT3* is associated with the regulation of *PD-L1* expression and the *PD-1* checkpoint pathway, which may inhibit T-cell activity, potentially through up-regulating the target genes *CD4* and *JUN*. The patients in the C2/C3 group showed the increased expression of *NR4A1* and *NR4A2*, which have been reported to be associated with suppression of anti-tumor immunity [24]. To further validate the regulatory relationships between TFs and their target genes, we first applied the ChIP-seq data from the ChIPBase v3.0 database to integrate experimentally determined binding [25,26]. The results showed that *IRF1*, *IRF9* and *STAT1* had experimentally determined binding at promoter regions of *CD8A*, *HLA-A*, *HLA-E*, *TAP1* and *PDCD1* (Figure 5D). Moreover, binding sites of *STAT3* and *JUN* were identified in both the promoter regions of *PD-L1* and *CXCL9*, respectively. In addition, we assessed the expression correlations between TFs and their target genes across the C1–C3 clusters using TCGA-KIRC data (Figure S6G). The expression level of *IRF1*, *IRF9* and *STAT1* was significantly positively correlated with *CD8A*, *HLA-A*, *HLA-E*, *TAP1* and *PDCD1* in the C1 cluster. These findings provide independent evidence supporting the predicted TF–target regulatory relationship identified in our gene regulatory networks.

In the myeloid-cell-associated GRN, patients in the C1 group showed increased activity of *STAT1* (FDR = 4.21 × 10^−158^), *IRF7* (FDR = 2.69 × 10^−97^) and *NR1H3* (FDR = 2.53 × 10^−90^) regulons (Figure S6C,D). For example, *STAT1*, *IRF7* and target gene *CXCL10* showed up-regulated expression in the C1 group, which might impact immune function [27]. In addition, *STAT1* and its target gene *S100A8* were up-regulated in the C1 group, which may contribute to the induction of myeloid-derived suppressor cells (MDSCs) and CD8+ T-cell exhaustion in multiple tumors [28]. *NR1H3* and its target genes influenced myeloid cell differentiation in the C1 group, which has been reported to be important for macrophage activation [29]. Furthermore, up-regulated *FOS* in the C2/C3 group impacted the MAPK signaling pathway, potentially through the down-regulated expression of target genes *HSPA1B* and *HSPA1A*, which contribute to the proliferation and differentiation of tumor cells [30,31]. *STAT1* and *FOS* had experimentally determined binding at promoter regions of *S100A8*, *HSPA1B* and *HSPA1A*, respectively (Figure 5D). Correspondingly, *STAT1* expression exhibited a significant positive correlation with *CXCL10* in the C1 cluster (Figure S6G).

**Figure 6:**
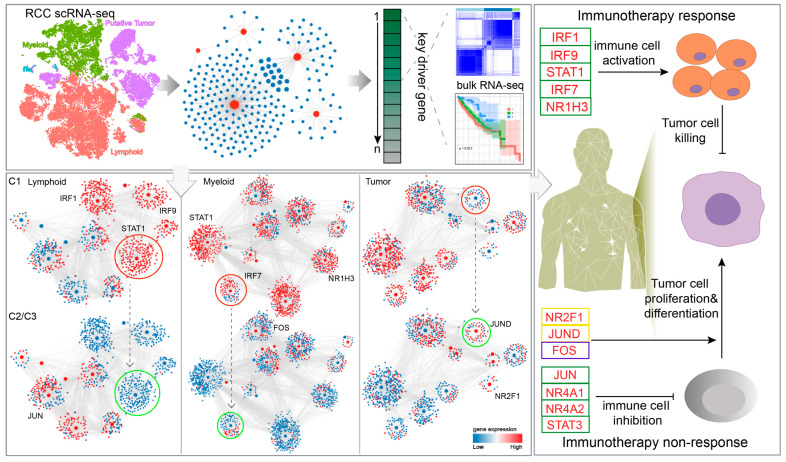
Overview of this study.

In the tumor-cell-associated GRN, patients in the C2/C3 group showed increased activity of *JUND* (FDR = 3.97 × 10^−277^) and *NR2F1* (FDR = 4.56 × 10^−283^) regulons (Figure S6E,F) and expression of *IL-6*. Functional enrichment analysis showed that up-regulated regulons in C2/C3 patients were mainly enriched in MAPK, PI3K-Akt, HIF-1 and Rap1 signaling pathways. In the C2/C3 group, the up-regulated *NR2F1* and the down-regulated target gene *TF* may be related to the mediation of the *HIF-1* signaling pathway, which could contribute to the hypoxic state and immune escape in C2/C3 patients [32]. Elevated expression was observed for *JUND* and its target gene *PDGFR* in the C2/C3 group, which have been implicated in PI3K-Akt pathways and could contribute to cancer proliferation, metastasis, invasion, and angiogenesis [33]. Taken together, these results suggest that immune-related candidate driver genes are associated with distinct regulatory programs across patient clusters, with C1 exhibiting an immune-active but complex microenvironment, while C2 and C3 are characterized by immunosuppressive and tumor-promoting features, potentially contributing to differential immunotherapy responses (Figure 6).

## 3. Discussion

With the advancement of scRNA-seq technology, the deep understanding of intra-tumoral heterogeneity is gained at the single-cell level [34]. In this study, specific GRNs of lymphoid cells, myeloid cells, and tumor cells were constructed to dissect immune-related candidate driver genes by integrating network centrality indices and immune-related genes. Here, 25 immune-related candidate driver genes were identified from three GRNs, which may be key factors and potential biomarkers for tumor progression and immunotherapy. Although several immune-related candidate driver genes, such as *STAT1*, *IRF1*, *JUN*, *FOS* and *STAT3*, are well-established regulators in cancer biology, the integration of genome-wide CRISPR-Cas9 screening data and immunotherapy cohorts enabled us to further reveal a set of potentially novel driver genes in RCC, including *JUND*, *TNFAIP3*, *NR4A2*, *RARA*, *ENG*, *NR2F1* and *NR2E1*. These genes may play important roles in tumor progression, immune microenvironment regulation, and response to immunotherapy, thereby providing new insights into RCC-specific regulatory mechanisms. While GRN-based computational inference can prioritize candidate driver genes with potential biological significance, experimental validation is still required to establish their causal driver activity.

Importantly, immune-related candidate driver genes can effectively stratify patients into clusters with distinct prognoses. Among them, the C1 group displayed a unique phenotype characterized by enhanced immune activation signatures alongside more advanced clinicopathological features and poorer overall survival in TCGA cohort. In addition, C1 showed significantly lower TIDE scores and higher TMB and somatic rates, but the opposite was true for C2 and C3. Further analyses in independent immunotherapy cohorts demonstrated that higher C1 scores were associated with improved treatment response and favorable survival trends, particularly when combined with low C2 or C3 scores. These findings indicate C1 may represent a candidate group of more aggressive tumors but benefit from immunotherapy, while C2 and C3 may be candidate groups with poor immunotherapy efficacy. Given the complexity of immunotherapy response, cluster C1 as the candidate responder group for immunotherapy is a computational hypothesis requiring clinical validation.

The construction of GRNs is helpful to elucidate the regulatory mechanism in the tumor microenvironment [35]. The regulatory relationships are verified through ChIP-seq data and published studies. *IRF1*, *IRF9* and *STAT1* activated the expression of target genes to regulate the immune-response-related functions, thereby improving the efficacy of ICI therapy [36]. *STAT3* and *FOS* regulated the expression of target genes to impact the tumor progression and immunosuppression-related functions, further alleviating tumor immunosuppression [37]. Importantly, our study provides several novel insights into TF–target regulatory relationships. The C1 cluster was characterized by *IRF7-CXCL10*, *IRF9-TAP1* and *IRF9-CD8A*, suggesting an immune-activated transcriptional program potentially linked to enhanced responsiveness to immunotherapy. The regulatory relationships of *NR2F1-TF*, *JUND-PDGFR*, *STAT3-CD4* and *JUN-CXCL9* were preferentially observed in the C2/C3 cluster, indicating a potential regulatory mechanism associated with hypoxia, immune evasion, and tumor progression in RCC. However, GRN inference relies on the regulatory function and expression levels of TFs [38]. Although we incorporated additional supporting evidence, including ChIP-seq data and expression correlation analyses in independent cohorts, we still cannot fully encompass the complexity of the tumor microenvironment. In the future, the regulatory relationships between TFs and potential target genes require experimental validation in vitro and in vivo in order to clarify their roles in RCC progression and immunotherapy response. Furthermore, the continuous emergence of large-scale scRNA-seq data from RCC patients receiving immunotherapy will facilitate more comprehensive validation and extension of our findings in future studies.

## 4. Materials and Methods

### 4.1. Data Collection and Preprocessing

Single-cell RNA sequencing data for RCC were retrieved from the Single Cell Portal database (https://singlecell.broadinstitute.org/single_cell, accessed on 14 December 2024), encompassing five patients who received immunotherapy (patient identification: P55, P915, P913, P906 and P912) and three patients who did not receive systemic treatment (patient identification: P76, P90 and P916). Among them, two were classified as responders to immunotherapy (patient identification: P55 and P915) and two as non-responders (patient identification: P913 and P906). The standard workflow based on anchoring in R package Seurat (v 5.4.0) was applied to integrate the scRNA-seq dataset, thereby correcting the differences among the samples [39]. Subsequently, Seurat was used for quality control, feature selection, dimension reduction, clustering and differential expression analysis. Marker genes from Bi et al. were used to identify the cell subtype of each cluster [12]. R package infercnv (v 3.20) was applied to identify malignant tumor cells (https://github.com/broadinstitute/inferCNV, accessed on 20 January 2025). TCGA-KIRC data were downloaded from The Cancer Genome Atlas (TCGA) database (https://portal.gdc.cancer.gov, accessed on 14 December 2024), including gene expression and clinical data of 530 RCC samples. The MuTect2-treated mutation dataset of TCGA-KIRC was retrieved from the UCSC database (http://xena.ucsc.edu/, accessed on 14 December 2024), with a total of 336 samples and 26,693 mutations. The validation set with 55 samples (GSE167573) was obtained from the GEO database (https://www.ncbi.nlm.nih.gov/geo/, accessed on 20 December 2024) [40]. A total of 2013 immune-related genes were from the “Immune System Process” term in GO and the “Immune System” event in Reactome and were acquired from the ImmPort database [41]. An RCC immunotherapy dataset was obtained from Braun et al., including RNA-seq data and clinical information of 311 patients who received PD-1 blocking immunotherapy (RCC-Braun2020) [42]. The immunotherapy dataset IMvigor210 for bladder cancer patients was used to explore the impact of immune candidate driver genes on the response to immunotherapy, including RNA-seq data and clinical information of 348 patients [43].

### 4.2. Gene Regulatory Network Inference

We used pySCENIC to infer gene regulatory networks (GRNs) separately for lymphoid, myeloid, and tumor cell populations based on scRNA-seq data [44]. First, the gene expression matrix (rows represent genes and columns represent the cells) was used as input. Second, the interactions of TF and target genes were inferred using GRNBoost2. Third, based on the interactions of TF and target genes, and cisTarget Human motif database v9 (https://resources.aertslab.org/cistarget/motif2tf/motifs-v9-nr.hgnc-m0.001-o0.0.tbl, accessed on 22 January 2025), co-expression modules were refined using cisTarget with default parameters, including thresholds of 0.75 and 0.90, top_n_targets = 50 and modules with more than 20 genes. Fourth, regulons (the regulatory network of TFs and target genes) were identified with df2regulons using default parameters, including the rank threshold of 5000 and the normalized enrichment score threshold of 3.0. Finally, regulon activity in individual cells was quantified using the AUCell algorithm with AUC threshold of 0.05. Cytoscape 3.9.1 was used to visualize the network.

### 4.3. Identification of Immune-Related Candidate Driver Genes in Gene Regulatory Network

To measure the importance of the network nodes within the GRN, the R package igraph (v 2.2.1) was used to separately compute the centrality indices of the network, covering degree, betweenness, eigenvalue, PageRank and closeness. Order statistics were applied to fuse these centrality indices for prioritizing node genes in the network, and this is defined as the Q statistic for each gene [45]. The Q statistic was calculated using the following formula:

$$\begin{array}{l} V_{k}=\sum_{j=1}^{k}{\left(-1\right)}^{j-1}\frac{V_{k-1}}{j!}r_{N-k+1}^{j}\\ Q(r_{1},r_{2},\ldots ,r_{i}\ldots ,r_{N})=N!V_{N} \end{array}$$

Here, $r_{i}$ denotes the rank ratio corresponding to the centrality index i. N represents the total number of centrality indices. The recursive calculation is initialized with $r_{0}=0$ and $V_{0}=1$.

### 4.4. Identification of Patient Clusters

Based on the expression profiles of immune-related candidate driver genes, the R package ConsensusClusterPlus (v 1.72.0) was employed to construct the consensus patient clusters. An intra-group proportion (IGP) algorithm was applied to evaluate the consistency of patient cluster between the TCGA-KIRC cohort and GSE167573 [46,47].

### 4.5. Functional Enrichment and Gene Set Level Analysis

The R package clusterProfiler (v 4.16.0) was used to perform GO and KEGG enrichment analysis for immune-related candidate driver genes and regulators in GRNs [48]. The hallmark pathways were downloaded from the MSigDB database, including 50 gene sets (http://www.gsea-msigdb.org/, accessed on 15 February 2025). The enrichment abundance of gene sets in different patient clusters was calculated by GSVA and ssGSEA [49].

### 4.6. Evaluation of the Immunotherapy Response

The TIDE algorithm was used to predict the immunotherapy response of patients in the C1–C3 clusters [50]. Patients with an elevated TIDE score may not respond to immunotherapy, while those with a low TIDE score may respond positively to immunotherapy.

### 4.7. Binding of Transcription Factors at the Promoters of Target Gene

Based on prior studies, the ChIP-seq data from ChIPBase v3.0 was used to evaluate transcription factor binding events within the promoter regions ranging from −5 kb upstream to +1 kb downstream of the transcription start sites of target genes [25,26].

### 4.8. Statistical Analyses

To evaluate differences in continuous variables, the Wilcoxon rank-sum test and the Kruskal–Wallis test were applied for comparisons involving two groups and multiple (≥3) groups, respectively. The false discovery rate (FDR) correction was applied to correct the *p*-value. The log-rank test was used to evaluate differences between survival curves. Spearman correlation analysis was used to calculate the correlation. All statistical analyses were executed in R version 4.3.2.

## Data Availability

The data of this study are available from TCGA-KIRC (TCGA, https://portal.gdc.cancer.gov, accessed on 14 December 2024). The additional validation data in this article were obtained from GEO with the accession number GSE167573 (https://www.ncbi.nlm.nih.gov/geo/query/acc.cgi?acc=GSE167573, accessed on 20 December 2024). The MuTect2-treated mutation dataset of TCGA-KIRC was obtained from the UCSC database (http://xena.ucsc.edu/, accessed on 14 December 2024). Single-cell RNA sequencing data for RCC was obtained from the Single Cell Portal database (https://singlecell.broadinstitute.org/single_cell, accessed on 14 December 2024). The RCC immunotherapy dataset was obtained from Braun et al. [42]. The immunotherapy dataset IMvigor210 for bladder cancer patients was collected from R package IMvigor210CoreBiologies (v 2.0.0) [43]. The R code used in the analysis is available on GitHub (https://github.com/wangliTeam/data-and-code, accessed on 5 March 2026).

## Supplementary Materials

The following supporting information can be downloaded at: https://www.mdpi.com/article/10.3390/ijms27083467/s1.

## Abbreviations

RCC	renal cell carcinoma
GRN	gene regulatory network
scRNA-seq	single-cell RNA sequencing
ATC	adoptive T cell
DepMap	Cancer Dependency Map
ssGSEA	single-sample gene set enrichment analysis
NR	non-response
CR	complete response
PR	partial response
SD	stable disease
PD	progressive disease
TCGA	The Cancer Genome Atlas
TIDE	tumor immune dysfunction and exclusion
IGP	intra-group proportion
TMB	tumor mutation burden
ICD	immunogenic cell death
ICP	immune checkpoint
ICI	immune checkpoint inhibitor
TF	transcription factor
EMT	epithelial–mesenchymal transition
CNV	copy number variation
bulk RNA-seq	bulk RNA sequencing
