# Supplementary material for: Identification of Cell Subpopulation-Specific Driver Genes Reveals Ideal Candidates for Renal Cell Carcinoma Immunotherapy
Source: Int J Mol Sci. 2026 Apr 13;27(8):3467. doi: 10.3390/ijms27083467 (PMC13115843; doi:10.3390/ijms27083467)
Supplement: Supplementary file 1 [file ijms-27-03467-s001.zip › ijms-4221582-supplementary.pdf]

SUPPLEMENTARY INFORMATION

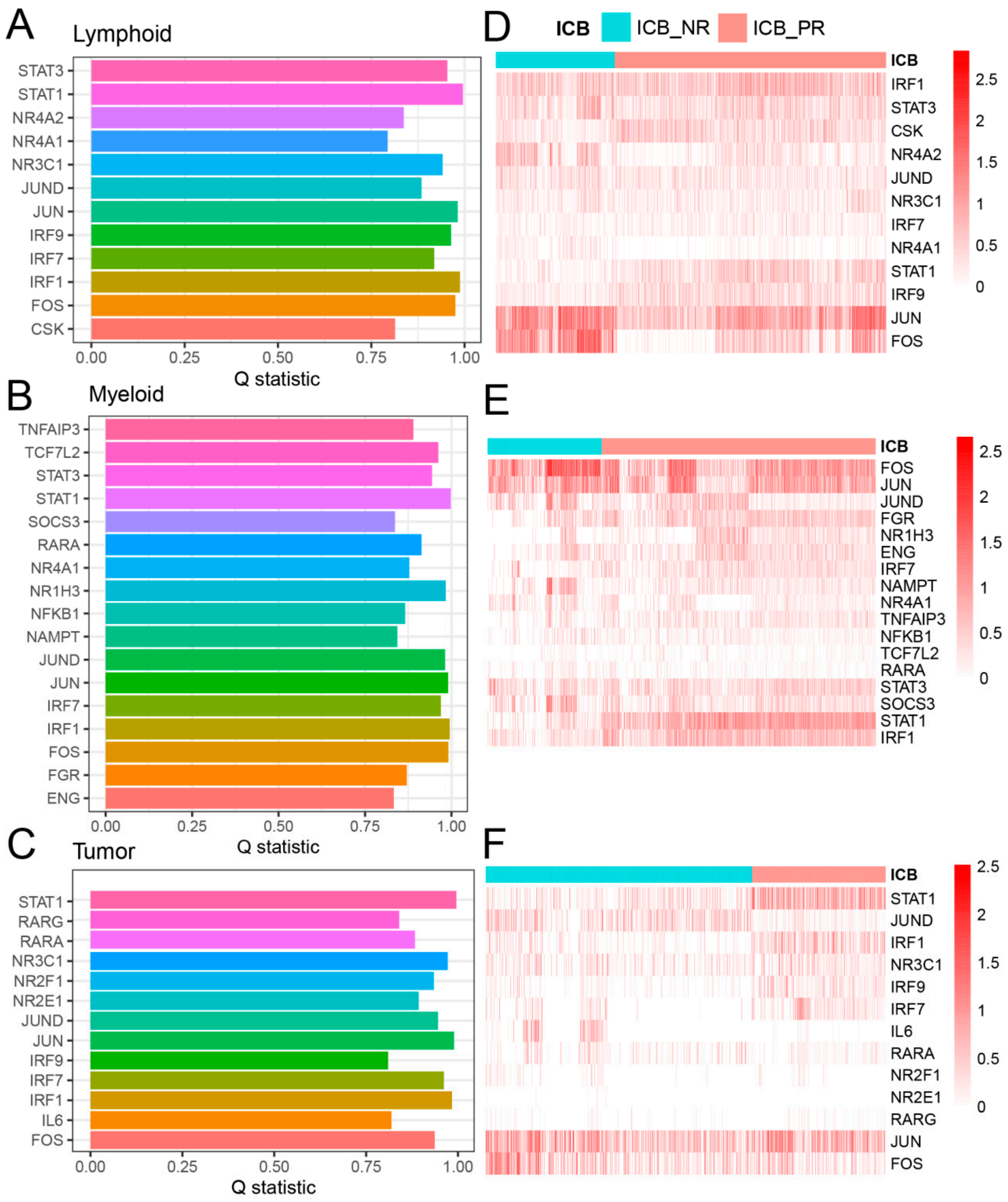

Figure S1: Q statistics and expression level of immune-related candidate driver genes. (A) Q statistics of immune-related candidate driver genes in gene regulatory networks of lymphoid cells. (B) Q statistics of immune-related candidate driver genes in gene regulatory networks of myeloid cells. (C) Q statistics of immune-related candidate driver genes in gene regulatory networks of tumor cells. (D) Expression level of immune-related candidate driver genes in lymphoid cells in the partial response (PR) and non-response (NR) immunotherapy patients. (E) Expression level of immune-related candidate driver genes in myeloid cells in the PR and NR immunotherapy patients. (F) Expression level of immune-related candidate driver

genes in tumor cells in the PR and NR immunotherapy patients.

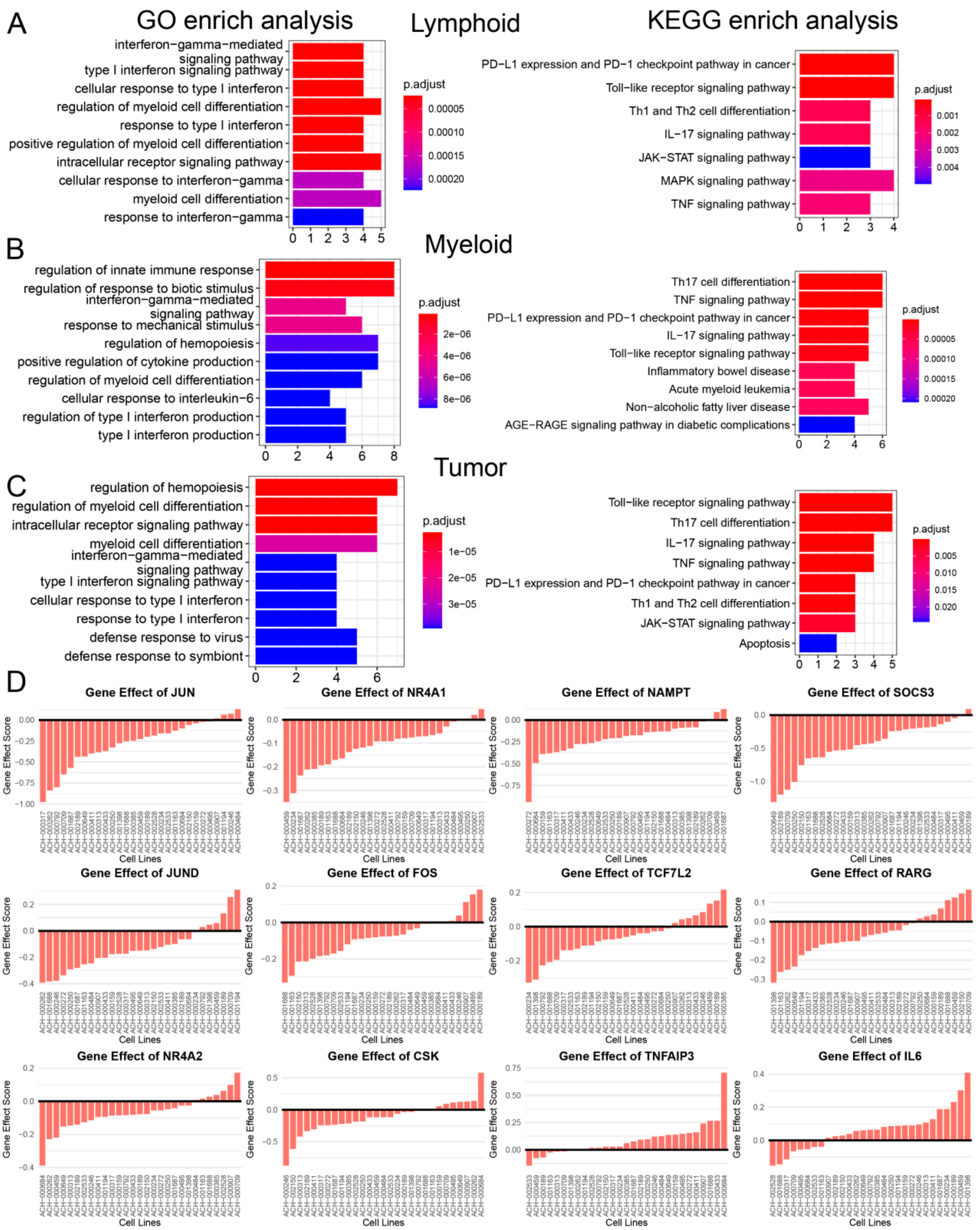

Figure S2: Function analysis of immune-related candidate driver genes. (A) GO and KEGG enrichment analysis of immune-related candidate driver genes identified in lymphoid cells. (B) GO and KEGG enrichment analysis of immune-related candidate driver genes identified in myeloid cells. (C) GO and KEGG enrichment analysis of immune-related candidate driver genes identified in tumor cells. (D) the functional impact of immune-related candidate driver gene knockdown in genome-wide CRISPR-Cas9 screening data.

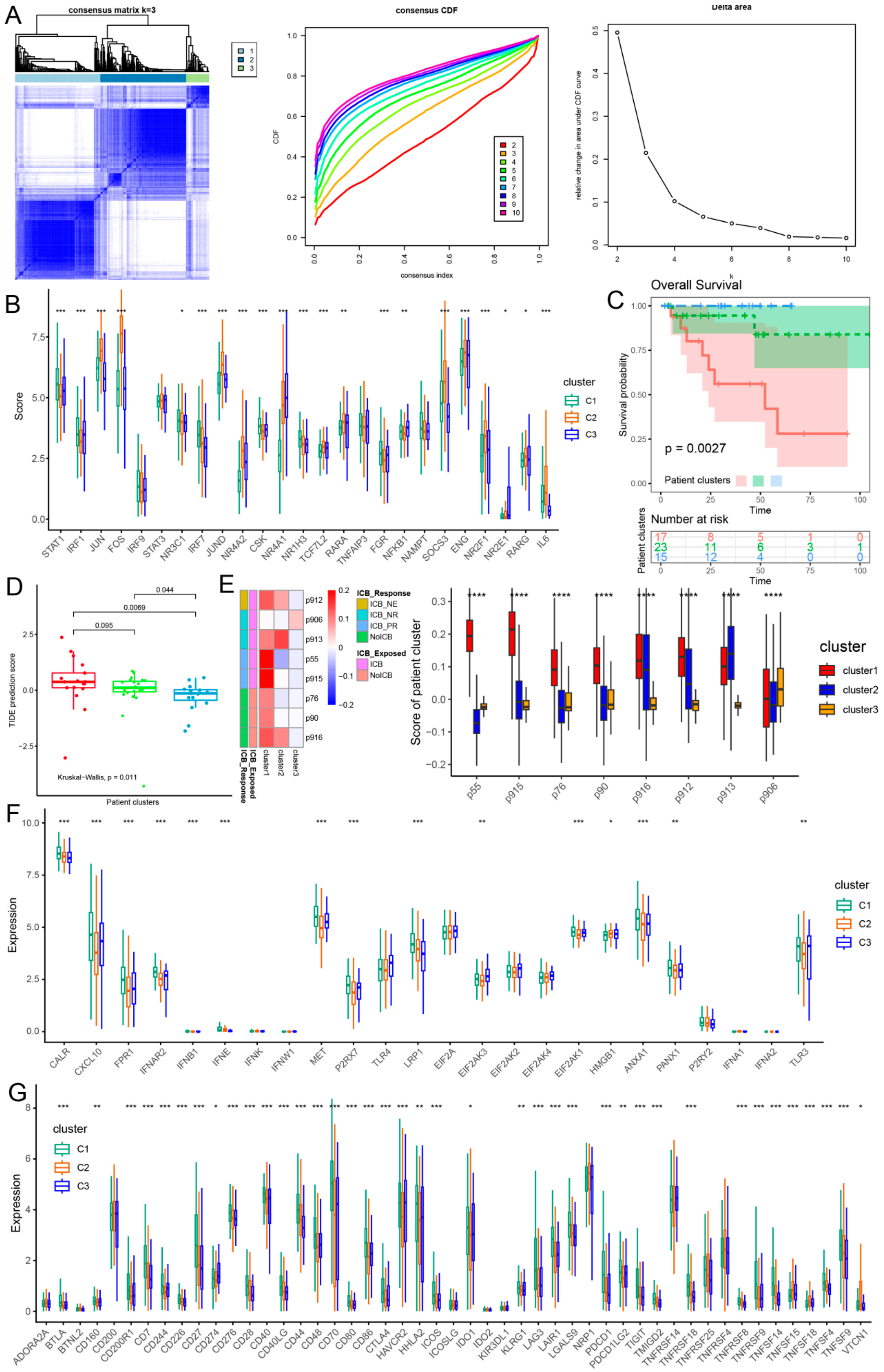

Figure S3: Characteristics among the C1-C3 patient clusters. (A) Sample clustering

heat map. Cumulative distribution function curve and delta area of driven-factors in TCGA cohort. (B) Differential expression of immune-related candidate driver genes among the patient clusters in TCGA cohorts.(C) Kaplan-Meier curves showing OS of patient clusters in GSE167573. (D) Distribution of TIDE scores among patient clusters in GSE167573. (E) In single-cell data, 8 patients were shown to enrich genesets of patient clusters. (F) Differential expression of ICD modulator genes among the RCC patient clusters in TCGA cohorts. (G) Differential expression of ICP genes among the RCC patient clusters in TCGA cohorts. The Kruskal-Wallis test and wilcoxon rank sum test were used for statistical significance. The false discovery rate (FDR) correction was applied to correct the p-value. ***: FDR< 0.001; **: FDR< 0.01; *: FDR< 0.05.

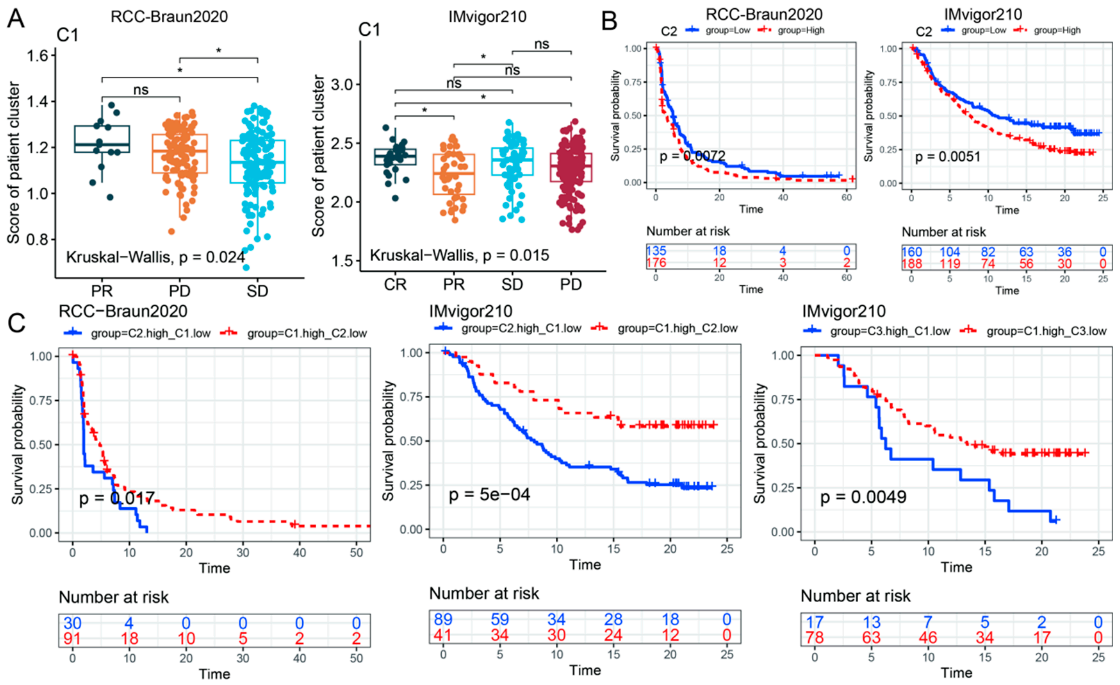

Figure S4: The clinical relevance of C1-C3 clusters in two immunotherapy cohorts. (A) The distribution of C1 scores among patients undergoing different immunotherapy treatments. (B) Survival analyses for low and high score of C2 patient groups in two immunotherapy cohorts (RCC-Braun2020 and IMvigor210) using Kaplan-Meier curves. (C) Survival analyses for both patient groups in two immunotherapy cohorts using Kaplan-Meier curves.

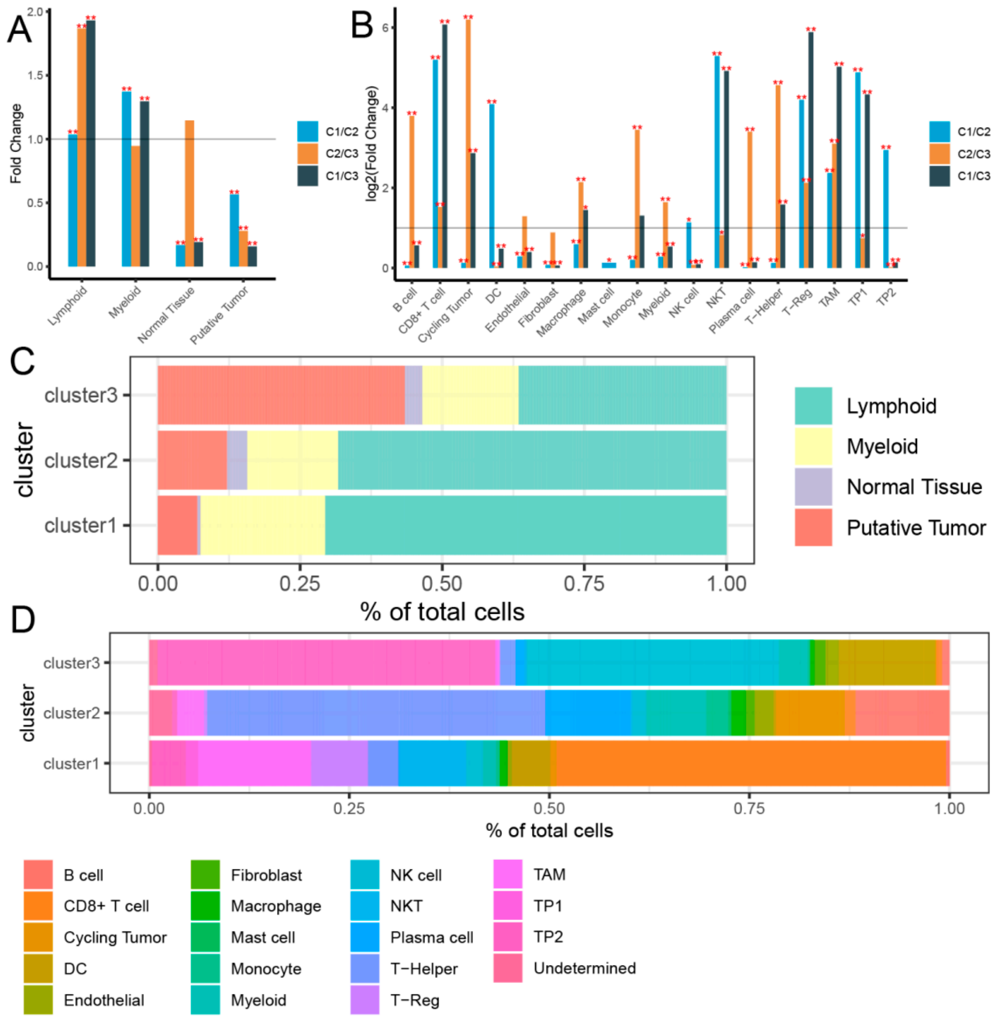

Figure S5: The distribution of cell types among the C1-C3 patient clusters. (A) Differences in cell lineages between immunotherapy patient clusters. (B) Differences in cell subtypes between immunotherapy patient clusters. (C) Distribution of four cell lineages among patient clusters. (D) Distribution of 18 cell subtypes among patient clusters.

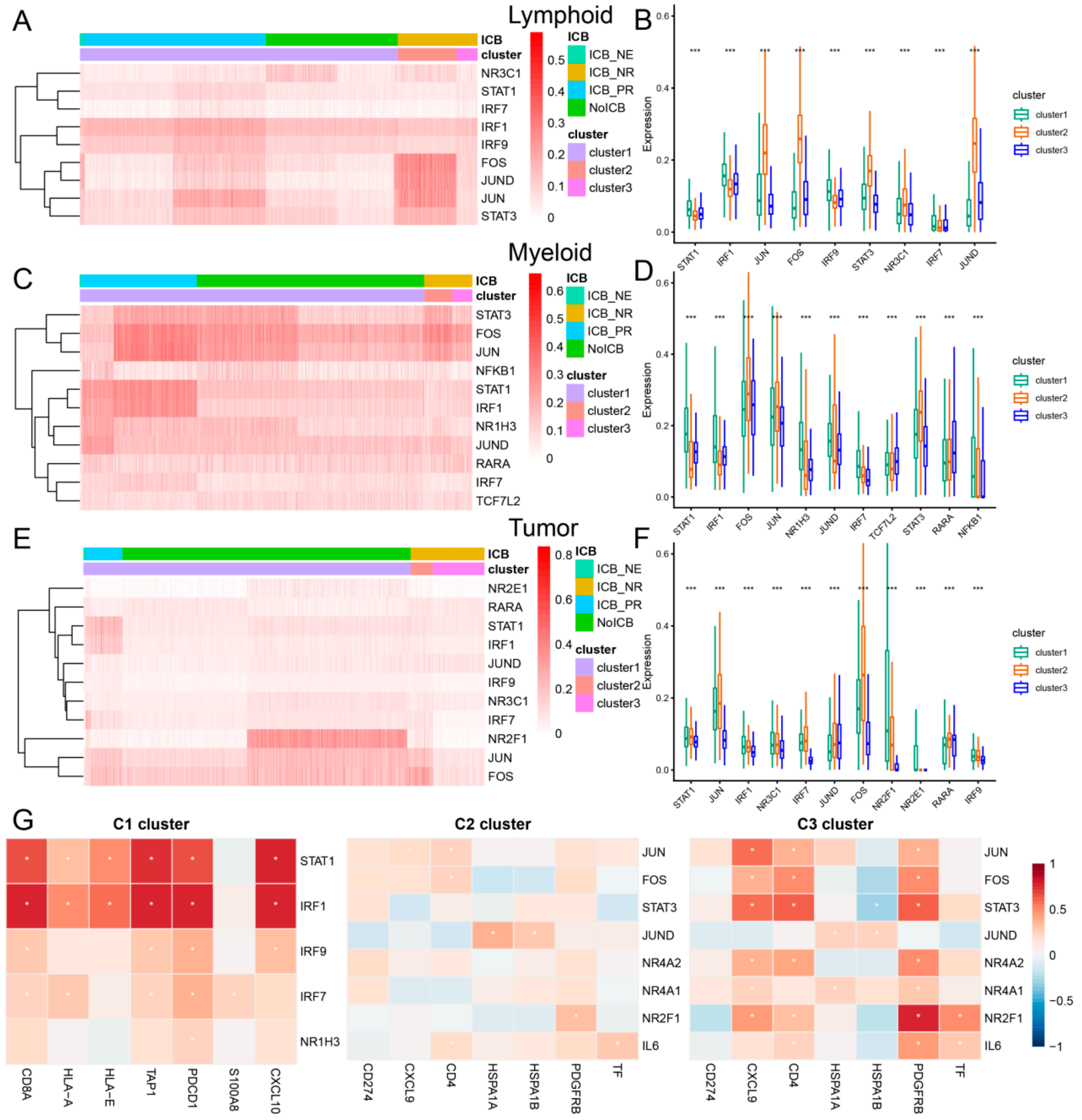

Figure S6: Single-cell network inference reveals the regulators of immunotherapy patient clusters. (A-B) Differences between clusters in the activity of the regulators of lymphoid cells. (C-D) Differences between clusters in the activity of the regulators of myeloid cells. (E-F) Differences between clusters in the activity of the regulators of tumor cells. The Kruskal-Wallis test was used for statistical significance. The false discovery rate (FDR) correction was applied to correct the p-value. ***: FDR< 0.001; **: FDR< 0.01; *: FDR< 0.05. (G) The expression correlations between TFs and their target genes across the C1-C3 clusters (*: |R|>0.2 and P<0.05).
